# Bridging the gap? Local production of medicines on the national essential medicine lists of Kenya, Tanzania and Uganda

**DOI:** 10.1186/s40545-022-00497-x

**Published:** 2023-01-30

**Authors:** Ayo-Oley Baldeh, Colin Millard, Allyson M. Pollock, Petra Brhlikova

**Affiliations:** 1 Institute of Population Health Sciences, Newcastle University, Newcastle Upon Tyne, NE2 4AX Tyne and Wear UK; 2 Ministry of Health, Kotu, The Gambia; 3 International Centre for Life, Newcastle University, Floor 2 Biomedicine West Wing, Newcastle Upon Tyne, NE1 7RU UK

**Keywords:** Local production, Essential medicines, Access to medicines, Public health, Medicine registration, Low-and-middle-income countries, East African Community, Kenya, Tanzania, Uganda

## Abstract

**Background:**

Essential medicines (EMs) are those that satisfy the basic healthcare needs of the population. However, access to EMs remains a global health challenge. The World Health Organization (WHO) and the East African Community (EAC) manufacturing plan 2017–2027 support local production of EMs as a strategy to improve access to medicines. The aim of this study was to determine for each therapeutic class on the national essential medicine lists (NEMLs) of Kenya, Tanzania and Uganda, the number of EMs produced in each country.

**Methods:**

In 2018, we analysed NEMLs and national drug registers (NDRs) in each country to identify local manufacturers and local products by EM status. For each local manufacturer we determined the number of EM products and individual EMs, and analysed EMs in each therapeutic class by registration status and whether produced locally.

**Results:**

There were nine companies manufacturing locally in Kenya, four in Tanzania and six in Uganda. Most local medicine products were non-EM products. Of the 946 locally produced products in Kenya, 310 were EM products; of the 97 locally produced products in Tanzania, 39 were EM products; and of the 181 locally produced products in Uganda, 100 were EM products. Many local EM products were duplicate. Only a small proportion of EMs on each NEML were produced locally: 21% (92/430) in Kenya, 5% (24/510) in Tanzania, and 10% (55/526) in Uganda. Kenya, Tanzania and Uganda had no local EM products in 13/32, 17/28 and 15/32 therapeutic classes, respectively. The proportion of EMs that were registered varied across the countries from 327 (76%) in Kenya, 269 (53%) in Tanzania, and 319 (60%) in Uganda.

**Conclusions:**

This study highlights the importance of auditing NDRs and NEMLs for local production to inform regional and national local manufacturing strategies. EMs should be prioritized for local production and drug registration to ensure that production is aligned with local health needs.

## Introduction

Access to essential medicines (EMs) remains a major global health challenge. The World Health Organization (WHO) estimates that 2 billion people are without access to EMs [1]. Local production of medicines is among a number of strategies to improve access to EMs in low- and middle-income countries (LMICs) [2].

Studies [3, 4] have highlighted the limited evidence linking local production of medicines to improved access. A 2011 WHO report identified the need for better alignment between industry and public health goals and proposed a framework based on national essential medicine lists (NEMLs) to guide the support of local production in LMICs [5]. There are no recent studies assessing the contributions of local production to medicine availability.

In East Africa, national support for local production is reinforced at a regional level through The East African Community (EAC), an intergovernmental organization, established by a Treaty in 2000 to enhance cooperation among its six member states: Burundi, Kenya, Rwanda, South Sudan, Tanzania and Uganda. The EAC agreement involves a customs union including free trade between member states and a common external tariff [6]. In 2015, the EAC took a major step toward the integration of member state pharmaceutical industries by harmonising the requirements for the registration of medicines [7].

As part of the region’s social and political integration, the EAC has developed its 2^nd^ Regional Pharmaceutical Manufacturing Plan 2017–2027 which sets out four high-level targets over the 10 year period for increasing local production, and 19 implementation indicators. While the plan states its commitment to EMs, only one indicator (indicator 11) refers to EMs, namely, to increase the proportion of EMs procured from EAC drug manufacturers to at least half of all EMs procured [8]. Moreover, the plan makes no reference to the WHO framework for local production, nor does it provide an analysis of NEMLs, registration of EMs, and whether produced locally.

There are three other indicators (indicators 16, 17, 19) relating to local production: reducing reliance on pharmaceutical imports from outside the EAC by 20%; promoting the expansion of product portfolios to meet the needs for over 90% of diseases; and having at least five companies that manufacture advanced pharmaceutical formulations, such as sustained release tablets, immune sera, layered tablets, and vaccines (Box 1). However, there are no data against which to measure progress for any of the four targets. The aim of this study was to determine for each therapeutic class on the NEMLs of Kenya, Tanzania and Uganda, the number of EMs produced locally.

Box 1 EAC pharmaceutical plan high level targets, baseline data and milestones **Target 1**

Reduce dependency on imports from outside the EAC from an estimated 70% in 2017 to 65% by 2021, 60% by 2025 and 50% by 2027 (indicator 17).

**Target 2**

Increase the percentage of disease conditions covered by product portfolio of EAC firms from an estimated 66% in 2017 to 75% by 2021, 80% by 2025 and 90% by 2027 (indicator 19).

**Target 3**

Increase percentage of EMs purchased by public procurement agencies from EAC manufacturers to 15% by 2021, 25% by 2025 and 50% by 2027 (indicator 11).

**Target 4**

Increase the number of firms producing APIs and higher value chain pharmaceuticals in the EAC region from 1 firm in 2017 to 2 by 2021, 3 by 2025, and 5 by 2027 (indicator 16).

## Background

Kenya, Tanzania and Uganda regard all companies registered and domiciled in the country as local manufacturers irrespective of ownership and level of manufacturing [8–10]. The level of manufacturing is characterized by three stages: primary, the manufacture of active pharmaceutical ingredients (APIs); secondary, the manufacture of complete dosage forms from raw materials and inactive substances; and tertiary, the packaging and relabelling of finished products [3].

### Kenya

In Kenya, the Pharmacy and Poisons Board is authorized under the Pharmacy and Poisons Act (Cap 244) to regulate the manufacturing, trade, and distribution of pharmaceutical products [8]. The first Kenyan NEML was established in 1981 and has been updated five times by the Ministry of Health (latest version is 2019). The government’s Kenya Medical Supplies Agency (KEMSA) is responsible for procuring medicines. However, due to budget constraints, many medicines on the NEML are not purchased; KEMSA procured 34% of the medicines listed on its NEML in 2010 [8]. Other procurers include the Mission for Essential Drugs and Supplies and the Procurement and Supply Chain Management Consortium. Kenya’s pharmaceutical market is the largest, fastest-growing market in the EAC, worth around USD 740 million in 2015 [11]. Local manufacturers produce both branded generic and generic medicines and accounted for approximately 30% of the domestic market in 2014 [11]. Secondary and tertiary manufacturing was reported in the country but there was no production of APIs [12]. India is the main supplier of raw materials and also accounted for nearly 40% of imported products in 2010 [8].

### Tanzania

The Tanzania Medicines and Medical Devices Authority (TMDA) is mandated by the Medicines and Medical Devices Act (Cap 219) to regulate the manufacturing, importation, distribution and sale of medicines, medical devices and diagnostics [10]. As the first African regulatory authority, TMDA was assessed by the WHO in 2018 as a well-functioning regulatory system for medicinal products [11]. Tanzania established its first NEML in 1991 and has had five updates, the latest is 2021. Medicines are supplied through the government's Medical Stores Department (MSD) and private organizations. The MSD is the main procurer of EMs in the country and provides medicines to the public sector and other organizations involved in healthcare provision. Tanzania’s pharmaceutical market was worth an estimated $400 million in 2015 and locally produced products constituted 12% of the overall market in 2014 [11]. Local manufacturers engage only in secondary and tertiary manufacturing[10], and like Kenya, raw materials are imported mainly from India [13].

### Uganda

The National Drug Authority (NDA) is Uganda’s drug regulatory agency under the National Drug Policy and Authority Act, Cap 206 [14]. The NDA controls the importation, exportation, and sale of drugs, including licence provision to pharmacies, wholesalers and local drug manufacturers. Uganda adopted its first NEML in 1991, with four updates, most recently in 2016. The National Medical Stores and the Joint Medical Stores procure and supply medicines as wholesalers to the public sector including non-governmental and faith-based organizations [14]. Approximately 20% of medicines were produced locally in Uganda in 2014, and its pharmaceutical market was worth around $450 million in 2015 [11]. Only secondary and tertiary-level production was reported in Uganda [14]. Raw materials for local production are imported mainly from China and India [9].

## Methods

### Research design

An audit of NDRs and NEMLs to ascertain registration of EMs, names of local manufacturers and their contribution to the availability of EMs and EM products.

### Data sources

The research was conducted in 2018 and drew on the most recent data sources at that time.

1. Country NDRs (accessed February 2018), listed 6151, 3956, and 3896 registered products for Kenya, Tanzania and Uganda, respectively. Medicines are listed by International Non-proprietary Name (INN), also known as the generic name, and the name of the corresponding registered product (branded, branded generic or generic). An individual medicine listed by its INN may correspond to numerous registered products due either to a company producing different formulations (dosage forms/strengths) of the medicine or multiple companies manufacturing versions of the same medicine.
2. Country NEMLs, version 2016 for Kenya and Uganda, and 2017 for Tanzania; accessed February 2018.

### Analysis

For each registered product at country-level, INN, product name, dosage form, product strength, registrant, name of manufacturer, and manufacturer’s country were extracted and entered in excel spreadsheets. Non-medicinal products (e.g., oxygen, plasma, platelets, red blood cells, condoms) and veterinary medicines were excluded. We identified EMs by comparing INNs with those on the NEML. The Tanzanian NDR lists products by manufacturer and country; the Ugandan NDR lists product by manufacturer and product origin; and the Kenyan NDR lists the registrant and whether the registrant is local or foreign. Having drawn up a list of local manufacturers from the register, we checked the local companies’ websites to exclude distributors and verify their country location. We looked at the EM status of all local/regional products and related these back to the corresponding medicine on the NEML.

## Results

### Local manufacturers of EMs and number of local products by EM status

We analysed the number of local manufacturers of medicines, number of registered local products and EM status of those products for each country.

Table 1 shows there were 19 companies listed as local drug manufacturers: 9 in Kenya with 946 registered products, 4 in Tanzania with 97 registered products and 6 in Uganda with 181 registered products. Some EMs have one or more locally produced products. Kenya had the highest number of EM products produced locally (310), followed by Uganda (100) and Tanzania (39). These products corresponded to 92 (21%), 24 (5%) and 55 (10%) individual medicines listed on the NEML for Kenya, Tanzania and Uganda, respectively.

**Table 1:** Number of local manufacturers *n*, registered products and number and proportion of locally produced EMs in Kenya, Tanzania and Uganda n, (%)

	Kenya	Tanzania	Uganda
Local manufacturers of medicines	9	4	6
Locally produced Medicine products	946	97	181
NEML products	310 (33)	39 (40)	100 (56)
Medicines on NEML	430	510	526
Locally produced EMs	92 (21)	24 (5)	55 (10)

### Registration status of EMs by therapeutic class and proportion produced locally

Using 2018 NDRs for each country, we analysed the number of EMs in each therapeutic class, by registration status and whether produced locally (Table 2).

**Table 2:** Number of EMs by therapeutic class, registration status, and proportion (%) produced locally in Kenya, Tanzania and Uganda from 2018 NDRs

Therapeutic class	Kenya			Tanzania			Uganda		
	EMs *n*	Registered EMs *n* (%)	Local EMs *n* (%)	EMs *n*	Registered EMs *n* (%)	Local EMs *n*	EMs *n*	Registered EMs *n* (%)	Local EMs *n* (%)
Therapeutic classes common to all three countries and with some locally produced essential medicines									
Anaesthetics	14	14 (100)	1 (7)	35	11 (30)	None	24	10 (42)	None
Medicines for pain and palliative care	17	17 (100)	11(61)	23	16 (69)	2 (9)	27	23 (85)	6 (22)
Anti-allergics and medicines used in anaphylaxis	6	6 (100)	3 (50)	6	6 (100)	2 (33)	7	7 (100)	2 (28)
Antiepileptics/anticonvulsants	9	8 (89)	3 (33)	7	6 (86)	None	8	6 (75)	1 (12)
Anti-infective medicines	90	77 (86)	33(37)	99	74 (75)	11(11)	110	82 (74)	24(22)
Anti-migraine medicines	3	3 (100)	1(33)	5	4 (80)	None	9	5 (55)	None
Anti-neoplastic and immunosuppressive medicines	52	38 (73)	2 (4)	43	19 (44)	None	43	19 (44)	1 (2)
Anti-parkinsonism medicines	3	1 (33)	1 (33)	4	1 (25)	None	1	None	None
Medicines affecting the blood	13	6 (46)	2 (15)	13	6 (46)	None	11	5 (45)	1 (9)
Cardiovascular medicines	21	18 (86)	10(48)	38	24 (63)	1 (3)	29	18 (62)	1 (3)
Dermatological medicines (Topical)	20	15 (75)	4 (20)	20	11 (55)	2 (10)	18	10 (55)	3 (17)
Disinfectants and antiseptics	5	3 (60)	2 (40)	5	1 (20)	1 (20)	8	4 (50)	1 (12)
Diuretics	5	4 (80)	None	4	3 (75)	None	4	4 (100)	1 (25)
Gastrointestinal medicines	10	9 (90)	5 (50)	28	13 (46)	1 (3)	18	14 (78)	3 (17)
Hormones, other endocrine medicines and contraceptives	22	15 (68)	2 (9)	31	18 (58)	1 (3)	28	20 (71)	1 (4)
Ophthalmological preparations	18	16 (89)	3 (17)	46	18 (39)	1 (2)	37	18 (49)	3 (8)
Peritoneal and haemodialysis solutions	None	n/a	n/a	None	n/a	n/a	None	n/a	n/a
Medicines for mental and behavioural disorders	20	14 (70)	4 (20)	25	13 (52)	None	26	16 (62)	1 (6)
Medicines acting on the respiratory tract	6	6 (100)	1 (17)	7	4 (57)	1 (14)	8	6 (75)	1 (12)
Solutions correcting water, electrolyte and acid–base disturbances	9	8 (89)	2 (22)	7	5 (71)	None	11	6 (54)	4 (36)
Vitamins and minerals	7	3 (43)	2 (28)	14	2 (14)	1 (7)	10	4 (40)	1 (10)
Therapeutic classes common to all three countries and with no locally produced essential medicines									
*Antidotes and other substances used in poisoning	15	2 (13)	None	9	1 (11)	None	17	5 (29)	None
*Blood products of human origin and plasma substitutes	7	4 (57)	None	7	None	None	4	None	None
*Diagnostic agents	2	None	None	2	2 (100)	None	6	None	None
*Immunologicals and vaccines	20	18 (90)	None	17	1 (6)	None	22	7 (32)	None
*Muscle relaxants (peripherally acting) and cholinesterase inhibitors	6	5 (83)	None	1	1 (100)	None	7	4 (57)	None
*Oxytocics and anti-oxytocics	6	5 (89)	None	6	6 (100)	None	4	3 (75)	None
*Ear, nose and throat medicines	7	4 (57)	None	8	3 (37)	None	13	11 (85)	None
Therapeutic classes unique to one or two countries and with no locally produced essential medicines									
Specific medicines for neonatal care (Uganda/Kenya)	5	2 (40)	None	X	X	X	4	3 (75)	None
Medicines for diseases of joints (Uganda/Kenya)	7	5 (71)	None	X	X	X	4	2 (50)	None
Nutrition (Uganda/Kenya)	4	1 (25)	None	X	X	X	6	2 (33)	None
Medicines for other conditions (Kenya)	1	None	None	X	X	X	X	X	X
Medicines for neurosurgical use (Uganda)	X	X	X	X	X	X	2	None	None
Total NEML medicines	430	327(76)	92(21)	510	269 (53)	24 (5)	526	314 (60)	55(10)

*Asterisk and italics denotes classes that had no local production in any country; *n/a* not applicable

The Tanzanian NEML has 28 therapeutic classes, while the Kenyan and Ugandan NEMLs have 32 therapeutic classes. The first 28 classes listed in Table 2 are common to all three countries. Peritoneal and Haemodialysis Solutions has no medicines listed on any of the NEMLs.

The number and proportion of EMs that were registered were 327 (76%) in Kenya, 269 (53%) in Tanzania, and 319 (60%) in Uganda. Kenya had 92 locally produced EMs across 19 drug classes, with no locally produced EMs for 13 classes. Tanzania had 24 locally produced EMs across 11 drug classes, with no locally produced EMs for 16 classes. Uganda had 55 locally produced EMs across 17 drug classes, with no locally produced EMs for 14 classes. Of the 28 classes common to all three countries, seven classes (marked by an asterisk in Table 2) had no local production in any country. The anti-infective class had the highest number of locally produced EMs with 33 produced in Kenya, 11 in Tanzania, and 24 in Uganda. The percentages of locally produced EMs varied across therapeutic classes with no local production for several classes.

### Regional import and export of local products, local essential products, and essential medicines in Kenya, Tanzania and Uganda

We searched Kenya, Tanzania and Uganda’s NDR for products registered by manufacturers in either of the other two countries. Since Kenya’s NDR does not list country of origin, we determined regional imports to Kenya using a list of local manufacturers identified in Tanzania and Uganda’s NDRs and checking for their products on Kenya’s NDR.

**Fig. 1:**
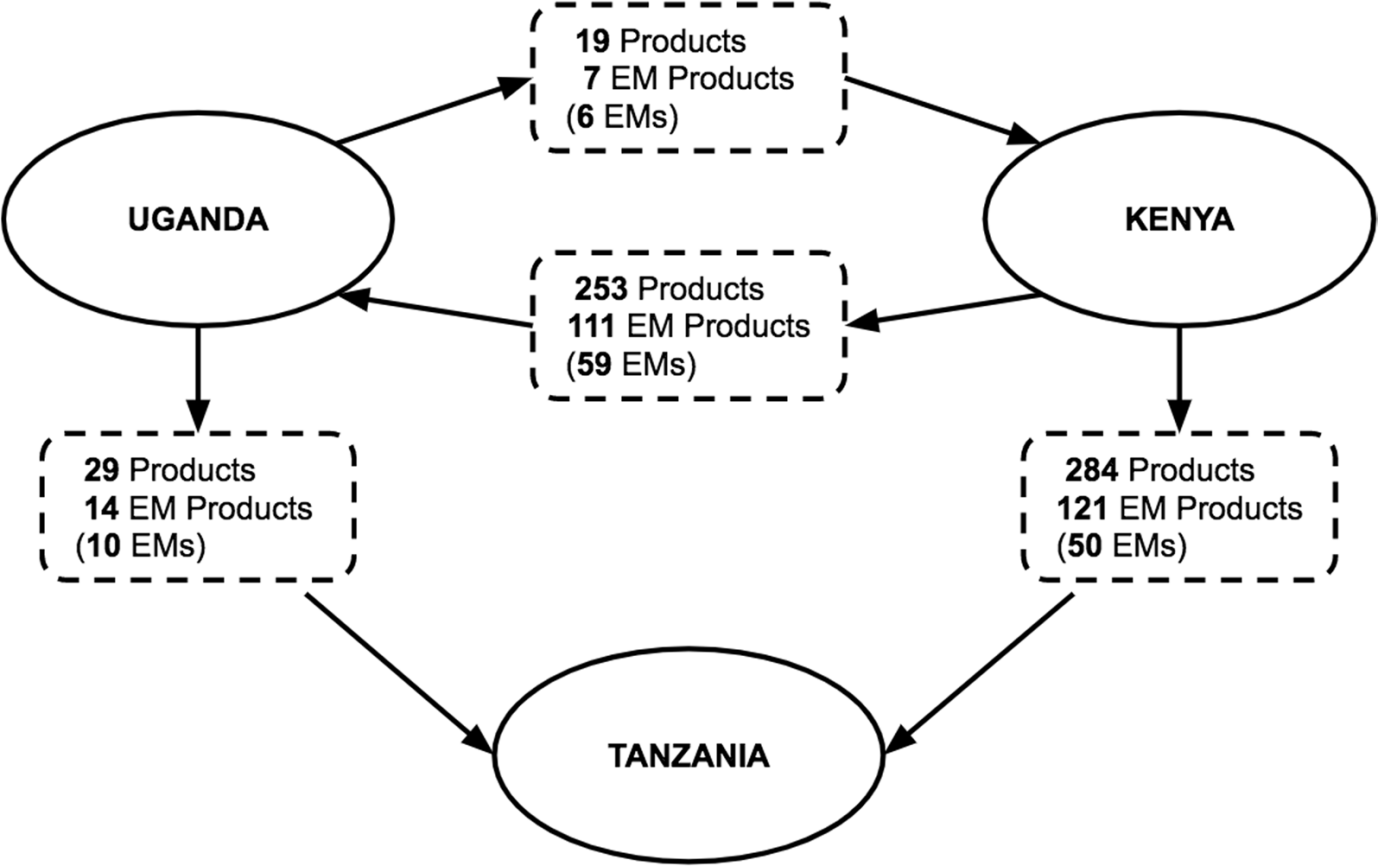
Import and export of local products, EM products, and EMs across Kenya, Tanzania and Uganda

Figure 1 shows the number of products produced locally and corresponding proportion of EM products and individual EMs. There was regional import of both EM and non-EM products from Kenya and Uganda to both neighbouring countries. None of the local manufacturers in Tanzania exported products to Kenya or Uganda.

A total of 19 products registered in Kenya were imports from Uganda; only 7 of these 19 (37%) products were listed on Kenya’s EML corresponding to 6 individual EMs. A total of 253 products registered in Uganda were imports from Kenya; 111 (44%) of these were listed on Uganda’s EML corresponding to 59 individual EMs. For products registered in Tanzania, a total of 284 products were imports from Kenya, of these 121 (43%) were listed on Kenya’s EML corresponding to 50 individual EMs; and 29 products were imports from Uganda, 14 (43%) of which were listed on Kenya’s EML corresponding to 10 individual EMs.

## Manufacturers and EM status of local products in Kenya, Tanzania and Uganda

For local products identified in the previous section we determined their EM status by comparing INNs with those on the NEML.

Table 3 shows the local manufacturers identified in each country along with the number of registered products, EM products, and individual EMs.

**Table 3:** Local manufacturers in Kenya, Tanzania and Uganda: number of registered products, number of registered EM products and as proportion (%) of all registered products, and number of individual EMs that products correspond to

Manufacturer name	Registered products *n*	Registered EM products *n* (%)	Registered EMs *n*
KENYA			
GlaxoSmithKline Kenya	12	0	0
Medisel	2	0	0
Benmed	23	6 (26)	4
Regal Pharmaceuticals	94	26 (28)	16
Dawa Limited	133	37 (28)	28
Biodeal Limited	95	38 (40)	30
Universal Corporation Limited	109	42 (38)	30
Lab and Allied	222	64 (29)	49
Cosmos Limited	256	97 (39)	66
Total	946	310 (33)	92
TANZANIA			
Keko Pharmaceuticals	7	5 (71)	5
Prince Pharmaceuticals	17	6 (35)	6
Zenufa Laboratories	19	8 (42)	8
Shelys Limited	54	20 (37)	17
Total	97	39 (40)	24
UGANDA			
Medipharm Industries	2	0	0
Kwality-Afro Asia Limited	1	1 (100)	1
Cipla Quality Chemicals	11	9 (82)	7
Abacus Parenteral	27	18 (66)	12
Kampala Pharmaceuticals	62	34 (55)	28
Rene Industries Limited	76	35 (46)	31
Total	181	100 (54)	55

### Kenya

Nine local manufacturers had registered products in Kenya. Cosmos Limited, DAWA Limited, and Lab and Allied had the highest number of registered products. Cosmos Limited produced the highest number of EMs (66), followed by Lab and Allied (49), Biodeal Limited (30) and Universal Corporation Limited (30). Biodeal, Universal and Cosmos had the highest percentage of EM products (40%, 39% and 38%, respectively). Two of the local manufacturers (GlaxoSmithKline Kenya and Medisel) did not have any EM products registered.

### Tanzania

Four local manufacturers had registered products in Tanzania. The multinational company (MNC) Shelys, registered the most products, followed by Zenufa Laboratories, Prince Pharmaceuticals and KEKO Pharmaceuticals. Shelys produced the highest number of EMs (17). Despite having the smallest product portfolio, KEKO pharmaceuticals had the highest percentage of EM products (71%).

### Uganda

Six local manufacturers had registered products in Uganda. Rene Industries, Kampala Pharmaceuticals and Abacus Parenteral had the highest number of registered products. Rene Industries Limited had the highest number of registered products and produced the highest number of EMs (31). Although Cipla Quality Chemicals had a relatively small portfolio, most of its products (82%) were EM products.

## Discussion

The WHO framework for local production requires that priorities for local production are determined by the country NEML [5]. This study demonstrates how data on local manufacturers and local products can be extracted from the NDR and compared with the NEML to inform targets and specify priorities for regional manufacturing plans. Previous studies [15, 16] have used a similar methodology to examine the registration status of EMs at the country level.

The study shows the extent to which local manufacturers produce EMs. In 2018 Kenyan manufacturers produced around a fifth of medicines on its NEML with a third of local products being EM products. In Tanzania, four local companies produced 5% of medicines on the NEML and two-fifths were EM products. In Uganda, 6 local manufacturers produced 10% of medicines on NEML with approximately half being EM products. Since 2012, Uganda has increased the number of EM products from 40 products corresponding to 32 unique EMs [16] to 100 products corresponding to 55 unique EMs in 2018. A comparable analysis has not been identified for Kenya or Tanzania.

The EAC manufacturing plan target of ‘at least half of medicines on the NEML to be procured from EAC drug manufacturers’ is ambitious; in 2018 local manufacturers in Kenya, Tanzania and Uganda produced 21%, 5% and 10% of medicines on their NEMLs, respectively. Moreover, there is significant under-registration of EMs on the NEMLs. Only 76%, 53% and 60% of medicines on the NEML were registered in Kenya, Tanzania and Uganda, respectively.

Supporting local production is a key priority in the pharmaceutical strategic plans of all three countries [17–19]. One of the incentives for local manufacturers is tax exemption on raw materials [11]. Restricting incentives to medicines on NEMLs could increase EM production. However, a recent study of prices and availability of locally produced and imported medicines in Ethiopia and Tanzania showed that the benefits of local production for patients is dependent on the national policy context [20].

The proximity of EAC markets facilitates exports between member states. The heavy reliance on imports from outside the EAC makes the target of reducing dependency on imports outside the EAC by 50% by 2027 difficult to achieve. Similarly, the target of primary manufacturing status for five companies producing APIs and higher value chain pharmaceuticals is a challenge, considering the current reliance on imports of APIs and advanced pharmaceutical formulations from non-EAC countries as well as the limited number of drug classes produced. Local production of EMs is important in the context of recent health emergencies. The United Nations Conference on Trade and Development (UNCTAD) recognizes that the current COVID-19 pandemic exposes the vulnerability of drug supply chains that rely on a few manufacturers for raw materials or finished products [21]. It found that drug manufacturers in the EAC were using less than 50% of their capacity due to raw material shortages and restrictions related to COVID-19 [21]. Therefore, reducing reliance on imports is a key step in strengthening public health security.

The wider benefits of local EM production include availability and lower prices for patients. A WHO/Health Action International survey in Tanzania found more availability of local products in rural areas when compared with imported products [22]. Some studies [23, 24] suggest that locally produced products are cheaper due to fewer mark-ups and shorter supply chains. Although a previous study [25] has questioned the necessity of local production in every country and shown that local production is not viable everywhere, this study shows that there is local production in Kenya, Tanzania and Uganda, and a focus on EMs specifically could ensure that the local industry contributes to EM availability.

## Limitations

The audit was done in 2018 using the current NDRs and corresponding NEMLs at the time.

## Conclusions

This study highlights the importance of auditing NDRs and NEMLs and local manufacturers products to inform regional and national pharmaceutical plans and strategies for increasing availability of EMs. In addition to facilitating the strategic selection of EMs for local production, regular audits would enable the EAC to monitor progress toward the targets in the pharmaceutical manufacturing plan and under-registration of EMs. Policymakers should assess manufacturing capacities and identify which EMs are suitable for local production and barriers to production. Future research could explore ways in which pharmaceutical companies could be incentivized to prioritize EMs to address regional public health need.

## Data Availability

The data used in this research is from the national drug registers and national essential medicines lists of each country and is available on their respective Ministry of Health websites.

## Abbreviations

API: Advanced pharmaceutical ingredient
EAC: East African Community
EM: Essential medicine
INN: International Non-proprietary Name
KEMSA: Kenya Medical Supplies Agency
LMICs: Low-and-Middle-Income Countries
MSD: Medical Stores Department
NDA: National Drug Authority
NDR: National Drug Register
NEML: National essential medicines list
TMDA: Tanzania Medicines and Medical Devices Authority
UNCTAD: United Nations Conference on Trade and Development
WHO: World Health Organization
